# Spatial transcriptomics deciphers the immunosuppressive microenvironment in colorectal cancer with tumour thrombus

**DOI:** 10.1002/ctm2.70112

**Published:** 2024-12-01

**Authors:** Heming Ge, Zhengda Pei, Zhongyi Zhou, Qian Pei, Cenap Güngör, Linyi Zheng, Wei Liu, Fengyuan Li, Jingxuan Zhou, Yao Xiang, Haiping Pei, Yuqiang Li, Wenxue Liu

**Affiliations:** ^1^ Department of General Surgery Xiangya Hospital Central South University Changsha China; ^2^ National Clinical Research Center for Geriatric Disorders Xiangya Hospital Central South University Changsha China; ^3^ Department of General Visceral and Thoracic Surgery University Medical Center Hamburg‐Eppendorf Hamburg Germany; ^4^ Graduate Collaborative Training Base of Hunan Cancer Hospital Hengyang Medical School University of South China Hengyang China; ^5^ Department of Endocrinology Endocrinology Research Center Xiangya Hospital Central South University Changsha China; ^6^ Department of Pathology People's Hospital of Longhua District Shenzhen China; ^7^ Department of Geriatric Medicine Xiangya Hospital Central South University Changsha China

Dear Editor,

Vascular tumour thrombus (TT), a prominent indicator of early metastasis associated with poor prognosis, classifies colorectal cancer (CRC) patients as high‐risk according to the National Comprehensive Cancer Network and the European Society for Medical Oncology^1^ guidelines. Regretfully, the mechanisms by which TT occurs and develops remain unclear. In this study, we identified a distinctive transcriptomic profile of immune cells with a compromised phenotype in the TT microenvironment, where CD4^+^ T cells as negative regulators directly signal to CD8^+^ T cells.

Note that, 6150 CRC surgery patients at Xiangya Hospital from 2014 to 2019 were retrospectively analyzed, of which 1321 cases behaved with pathological vascular TT (Figure 1A,B and Table S1). Survival data available for 5109 patients demonstrated a significant separation in overall survival (OS) between those with and without TT (Figure 1C and Figure S1).

**FIGURE 1:**
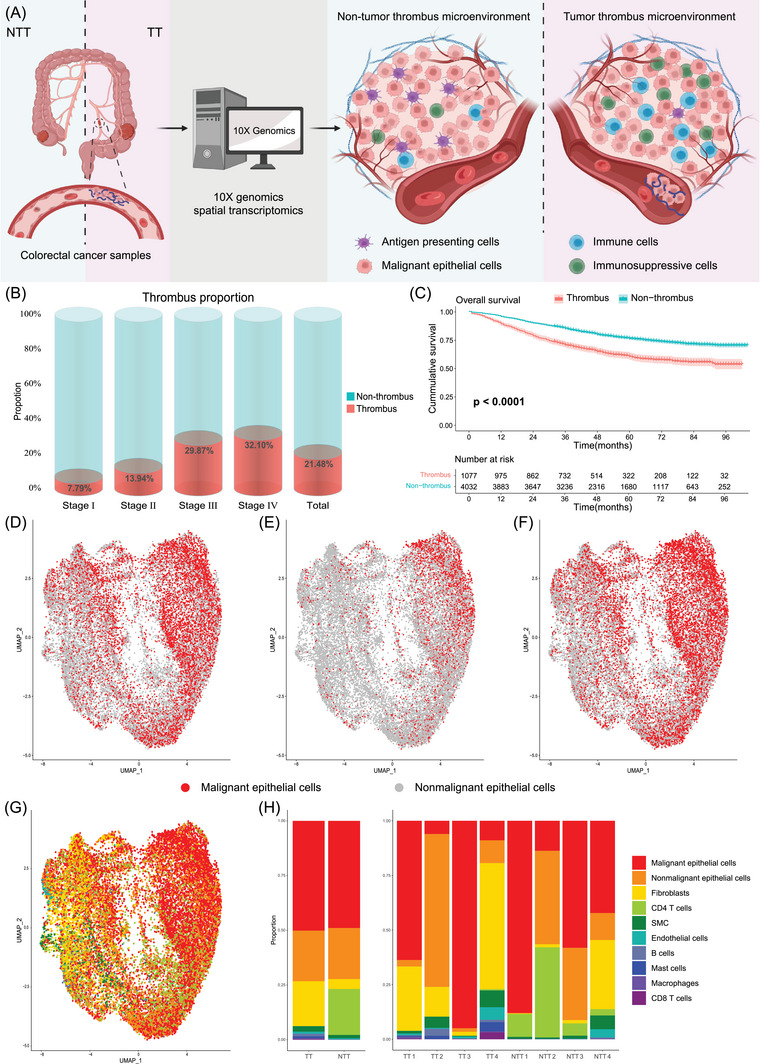
Overview of study design and spatial transcriptomics (ST) spots annotation. (A) Schematic representation of this study design. Tumour thrombus and non‐tumour thrombus tissues from colorectal cancer (CRC) patients were used for spatial transcriptomics RNA sequencing. (B) The proportion of patients with tumour thrombus across different stages of CRC. (C) Survival curve for CRC patients with and without tumour thrombus. (D–F) UMAP plots of malignant spots identified by CNA analysis (D), deconvolution analysis (E), and the combination of CNA analysis and deconvolution results (F). (G) ST spots annotation based on CNA analysis and deconvolution in carcinoma regions. (H) Proportion of different cell type spots within the carcinoma regions of TT and NTT samples. TT, tumour thrombus; NTT, non‐tumour thrombus; SMC, smooth muscle cells.

We performed spatial transcriptomics (ST) on four primary CRC samples with TT (TT 1–4; Figure S2A–D and Table S2) and compared them with four non‐TT (NTT) samples from dataset GSE226997^2^ (NTT 1–4; Figure S2E–H). Malignant epithelial cells were identified using CNA^3^ and deconvolution methods (Figure 1D–F and Figure S3A,B), while non‐malignant spots were classified into nine distinct clusters (Figure 1G,H and Figure S3C–K).^4^

The gene expression patterns of malignant epithelial cells were conserved in thrombus status (Figure S4A). Pathway enrichment related to angiogenesis and tumour growth was observed in malignant epithelial cells in TTs (Figure S4B,C and Tables S3 and S4). However, cancer‐promoting transcription factors revealed comparable expression activated in both TTs and NTTs (Figure S4D and Table S5). When categorising malignant epithelial cells into consensus molecular subtypes CMS1‐4 and iCMS2/3, we observed no significant differences in proportions or spatial distributions between TTs and NTTs (Figures S4E–J and S5A–E), nor in stem cell and epithelial‐mesenchymal transition scores (Figure S5F–I), indicating that the prognostic differences were predominantly driven by the microenvironment.

Spatial profiling showed an increase of CD8^+^ T cells and macrophages and a reduction of CD4^+^ T cells in TTs (Figure 2A–F and Figure S6A–F), confirmed by immunofluorescence (Figure 2G–J), with a heightened co‐localization of CD4+ and CD8+ T cells in TTs (Figure S7A–C).

**FIGURE 2:**
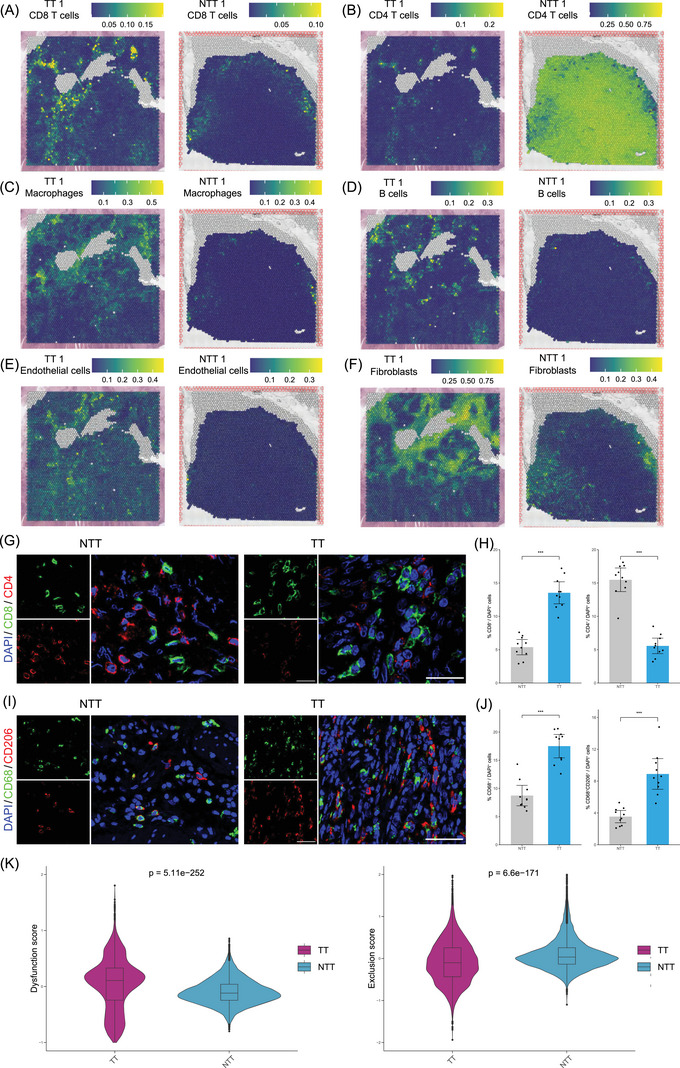
Spatial distribution of various cell types in TT and NTT samples. Spatial plots of CD8^+^ T cells (A), CD4^+^ T cells (B), macrophages (C), B cells (D), endothelial cells (E) and fibroblasts (F) in TT1 and NTT1 samples. (G) Immunofluorescence for CD8^+^ and CD4^+^ T cells on TT and NTT samples. Scale bar 25 µm. (H) Percentage of CD8^+^ and CD4^+^ T cells among total cells between TT and NTT groups. (I) Immunofluorescence for macrophages (CD68^+^) and M2 macrophages (CD68^+^CD206^+^) on TT and NTT samples. Scale bar 50 µm. (J) Percentage of macrophages and M2 macrophages among total cells between TT and NTT groups. (K) Differences in the TIDE dysfunction and exclusion scores were observed between TT and NTT samples. In TT samples, the lower exclusion score indicated that immune cells successfully infiltrated the tumour. However, the higher dysfunction score in TT samples suggested that, despite this infiltration, the immune cells had their functions suppressed, preventing them from effectively killing tumour cells. TT, tumour thrombus; NTT, non‐tumour thrombus; SMC, smooth muscle cells.

Contrary to expectations, the increased immune cells in TTs did not contribute to a better prognosis. We performed a functional analysis of immune cells to explore this paradox,^5^ indicating these immune cells with a state of dysfunction mostly within TTs albeit their abundance (Figure 2K).

Further analysis revealed a higher prevalence of immunosuppressive cells, including regulatory T cells (Tregs), M2 macrophages, monocytic myeloid‐derived suppressor cells (M‐MDSCs), polymorphonuclear MDSCs and tolerogenic dendritic cells in TTs (Figure 3A–E), suggesting a microenvironment dominated by immunosuppression. Immunostaining confirmed these findings (Figures 2I,J and 3G,H). Given that Tregs can inhibit antigen‐presenting cells, we examined the spatial distribution of conventional dendritic cells, which was reduced in TTs (Figure 3F), confirmed by immunofluorescence (Figure 3I,J). Additionally, classical antigen‐presenting molecules also exhibited lower expression levels in TTs (Figure S9). Chemokines, such as CCL5 and CXCL12, were notably elevated in TTs, which may partly explain the recruitment of both immune and immunosuppressive cells (Figures S8A,B and S9). Immune checkpoint genes like PDCD1 and CD274 were upregulated in TTs (Figures S8C,D and S9), validated by qPCR likewise (Figure 3K). Immunosuppressive genes and cytokines were also elevated in TTs (Figures S8E,F and S9).

**FIGURE 3:**
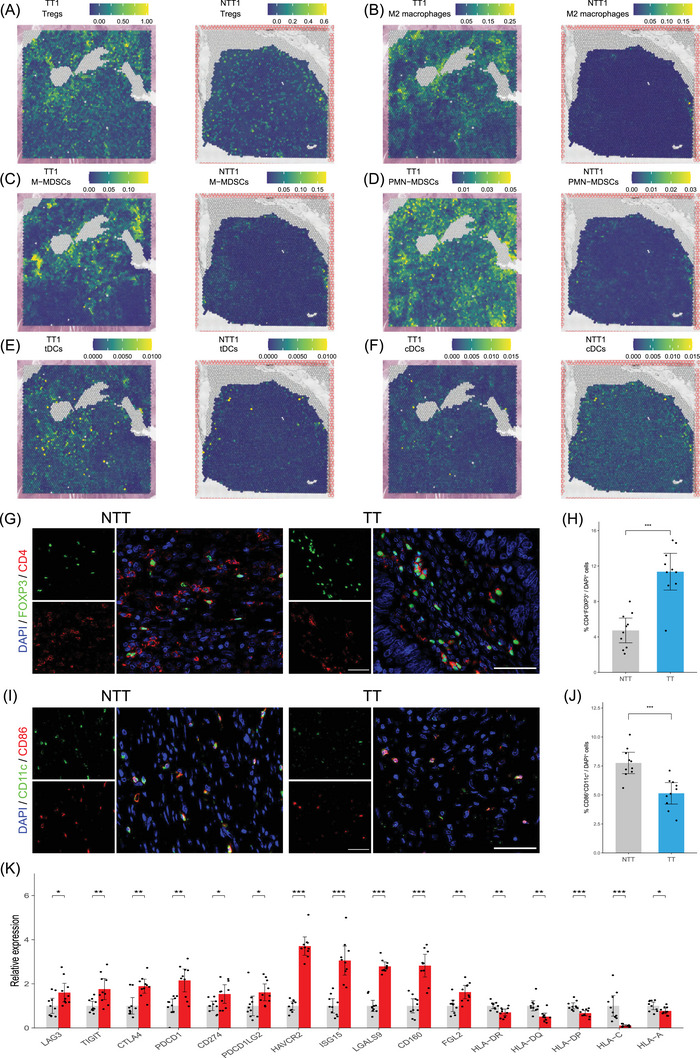
Immunosuppressive landscape of colorectal cancer (CRC) tumour thrombus. Spatial plots of regulatory T cells (Tregs) (A), M2 macrophages (B), monocytic myeloid‐derived suppressor cells (M‐MDSCs) (C), polymorphonuclear MDSCs (PMN‐MDSCs) (D), tolerogenic dendritic cells (tDCs) (E) and conventional dendritic cells (cDCs) (F) in TT1 and NTT1 samples. (G) Immunofluorescence for Tregs (CD4^+^FOXP3^+^) on TT and NTT samples. Scale bar 50 µm. (H) Percentage of Tregs among total cells between TT and NTT groups. (I) Immunofluorescence for cDCs (CD86^+^CD11c^+^) on TT and NTT samples. Scale bar 50 µm. (J) Percentage of cDCs among total cells between TT and NTT groups. (K) qPCR analysis of key immune checkpoint genes, immunosuppressive genes, and antigen‐presenting molecules in TT and NTT samples. TT, tumour thrombus; NTT, non‐tumour thrombus.

Intercellular communication^6^ showed more signalling events in NTTs, whereas TTs exhibited frequent interactions between CD8^+^ and CD4^+^ T cells (Figure S10A–D and Table S6). Receptor‐ligand pairs between CD8^+^ and CD4^+^ T cells, including CD80 ‐ CTLA4, CD86 ‐ CTLA4, LGALS9 ‐ CD45 and CD274 ‐ PDCD1, were identified as inhibitors of CD8^+^ T cell activation (Figure S11). Interestingly, these interactions were cell‐contact‐dependent rather than mediated by secreted factors, aligning with the spatial proximity between CD8^+^ and CD4^+^ T cells. Enrichment analysis showed a significant inhibitory pathway in CD8^+^ T cells within the TTs, whereas an obvious activation pathway was observed in NTTs (Figure S8G). This CD4^+^ T cell‐mediated inhibition was confirmed in thrombus samples from hepatocellular carcinoma, which demonstrated higher proportions of CD8^+^ T cells with suppressed activity due to CD4^+^ T cell interactions (Figure S12).^7^ Collectively, our findings suggested that CD4^+^ T cells exert an inhibitory effect on CD8^+^ T cells within TTs, resulting in immune cell dysfunction.

Differential gene expression analysis between TTs and NTTs identified 145 TT‐specific genes associated with poor prognosis (Table S7 and S8). TCGA‐CRC patients were stratified into high‐ and low‐score groups based on this gene signature, with the high‐score group exhibiting a worse prognosis (Figure 4A and Figure S13A,B). Two distinct datasets validated these findings (Figure S13C,D).^8^, ^9^ Notably, the high‐score group showed elevated immune scores (Figure 4B), accompanied by increased immunosuppressive Tregs and decreased activated/resting DCs and CD4+ T cells (Figure 4C and Figure S13E).^10^ These findings were consistent with our results from ST samples, substantiating the reduction of CD4^+^ T cells and antigen‐presenting cells and the simultaneous augment of Tregs within TTs. Additionally, TT score correlated positively with higher half inhibitory concentrations of key chemotherapeutic agents (Figure 4D), reinforcing the association with poorer clinical outcomes in patients with TT.

**FIGURE 4:**
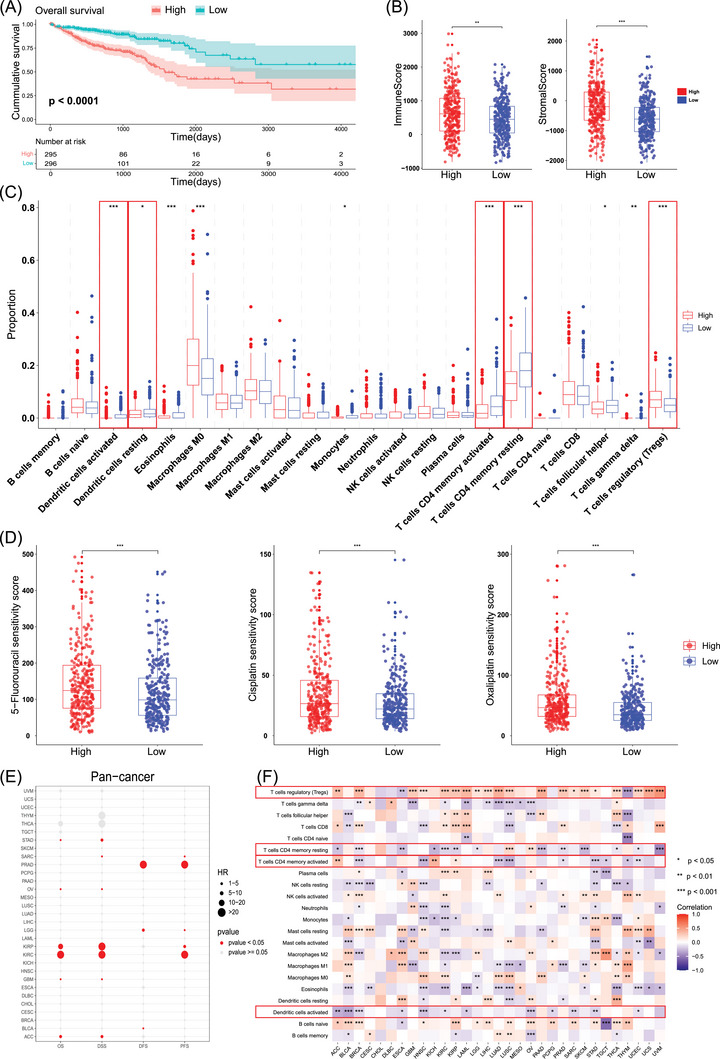
Construction of the tumour thrombus gene signature and pan‐cancer analysis. (A) Survival curve of high and low tumour thrombus score groups in TCGA‐CRC patients. (B) Comparison of immune and stromal scores between high and low tumour thrombus score groups. The high‐score group exhibited significantly higher proportions of immune and stromal cells compared to the low‐score group. (C) The proportions of 22 immune‐related cells between the tumour thrombus score groups by the CIBERSORT method. (D) Correlation analysis between tumour thrombus scores and chemotherapeutic drug resistance. (E) Pan‐cancer outcomes (OS, DSS, DFS and PFS) for tumour thrombus scores derived from 31 common cancer types from TCGA. (F) The relationship between tumour thrombus scores and immune‐related cell infiltration across 31 common cancer types from TCGA. OS, overall survival; DSS, disease specific survival; DFS, disease free survival; PFS, progression free survival; ACC, adrenocortical carcinoma; BLCA, bladder urothelial carcinoma; BRCA, breast invasive carcinoma; CESC, cervical squamous cell carcinoma and endocervical adenocarcinoma; CHOL, cholangiocarcinoma; DLBC, diffuse large B‐cell lymphoma; ESCA, esophageal carcinoma; GBM, glioblastoma multiforme; HNSC, head and neck squamous cell carcinoma; KICH, kidney chromophobe; KIRC, kidney renal clear cell carcinoma; KIRP, kidney renal papillary cell carcinoma; LAML, acute myeloid leukemia; LGG, brain lower grade glioma; LIHC, liver hepatocellular carcinoma; LUAD, lung adenocarcinoma; LUSC, lung squamous cell carcinoma; MESO, mesothelioma; OV, ovarian serous cystadenocarcinoma; PAAD, pancreatic adenocarcinoma; PCPG, pheochromocytoma and paraganglioma; PRAD, prostate adenocarcinoma; SARC, sarcoma; SKCM, skin cutaneous melanoma; STAD, stomach adenocarcinoma; TGCT, testicular germ cell tumors; THCA, thyroid carcinoma; THYM, thymoma; UCEC, uterine corpus endometrial carcinoma; UCS, uterine carcinosarcoma; UVM, uveal melanoma. *p < .05, **p < .01, ***p < .001.

Pan‐cancer analysis exhibited a significant correlation between the aforementioned gene signature and poor prognosis across several epithelial‐origin solid tumours (Figure 4E). Immunosuppressive Tregs showed a positive correlation with the TT gene signature, whereas both activated/resting CD4 memory T cells and activated DCs unveiled a negative correlation (Figure 4F). These findings aligned with findings from ST samples, indicating that an enhanced immunosuppressive state is a key feature of the TT microenvironment across diverse cancer types.

In conclusion, our study highlights that, despite their abundance in the TT microenvironment, immune cells demonstrate impaired anti‐tumour functionality due to upregulated immune checkpoints, immunosuppressive genes, and attenuated antigen presentation, which emphasizes the predominance of an immunosuppressive microenvironment. Given the reliance of current immunotherapies on the activation and efficacy of pre‐existing immune cells, our findings suggest that CRC patients with TT may benefit from enhanced immunotherapeutic strategies targeting immune cell reactivation.

## AUTHOR CONTRIBUTIONS

Heming Ge, Yuqiang Li and Wenxue Liu conceived and supervised the study. Zhongyi Zhou, Qian Pei, Fengyuan Li, Jingxuan Zhou and Haiping Pei contributed to the clinical data collection and patients’ follow‐up. Heming Ge, Zhengda Pei, Zhongyi Zhou, Qian Pei, Cenap Güngör and Yao Xiang contributed to the data analysis. Heming Ge, Zhengda Pei, Linyi Zheng and Wei Liu prepared figures and tables. Heming Ge, Zhongyi Zhou and Qian Pei wrote the first version of the manuscript. Cenap Güngör, Yuqiang Li and Wenxue Liu reviewed and edited the manuscript. All authors read and approved the final manuscript.

## CONFLICT OF INTEREST STATEMENT

The authors declare no conflict of interest.

## FUNDING INFORMATION

This study was supported by the National Natural Science Foundation of China (82273077), the Natural Science Foundation of Hunan Province (2022JJ40799) and the Medical Research Project of Longhua District Medical Association (2023LHMA22).

## ETHICS STATEMENT

This study was approved by the Ethics Committee of the Xiangya Hospital of Central South University (No. 2024020206).

## PATIENT CONSENT STATEMENT

All human tissue samples were obtained from patients with written informed consent.

## Supporting information



Supporting Information

Supporting Information

Supporting Information

## Data Availability

The raw data of spatial transcriptomics RNA sequencing of four TT samples were uploaded to the Genome Sequence Archive (GSA, https://ngdc.cncb.ac.cn/gsa‐human) of the National Genomics Data Center with accession number HRA010942. The raw data of spatial transcriptomics RNA sequencing of four NTT samples had been previously deposited in Gene Expression Omnibus (GEO, https://www.ncbi.nlm.nih.gov/geo/) repository with accession number GSE226997.^2^ The scRNA‐seq dataset reused in this study are available in the GEO database under accession number GSE132465.^4^ The normalized gene expression data of colon adenocarcinoma and rectum adenocarcinoma were obtained from TCGA data portal (https://www.cancer.gov/ccg/research/genome‐sequencing/tcga). The bulk RNA‐seq datasets of CRC patients were downloaded from GEO, including GSE39582^8^ and GSE38832.^9^ The bulk RNA‐seq datasets for pan‐cancer analysis were downloaded from the TCGA data portal.
