# Supplementary material for: Spatial transcriptomics deciphers the immunosuppressive microenvironment in colorectal cancer with tumour thrombus
Source: Clin Transl Med. 2024 Dec 1;14(12):e70112. doi: 10.1002/ctm2.70112 (PMC11608866; doi:10.1002/ctm2.70112)
Supplement: Supplementary file 1 — Supporting Information [file CTM2-14-e70112-s001.docx]

Supplementary materials and methods

**Spatial Transcriptomics Deciphers the Immunosuppressive Microenvironment in Colorectal Cancer with Tumor Thrombus**

Heming Ge, Zhengda Pei, Zhongyi Zhou, Qian Pei, Cenap Güngör, Linyi Zheng, Wei Liu, Fengyuan Li, Jingxuan Zhou, Yao Xiang, Haiping Pei, Yuqiang Li, Wenxue Liu

**This file includes:**

Supplementary materials and methods

**Supplementary Materials and Methods**

**Tumor thrombus clinical data**

We retrospectively collected clinical data from patients who underwent colorectal cancer surgery between January 2014 and December 2019 at Xiangya Hospital, Hunan Province, China based on the following inclusion and exclusion criteria. A total of 6,150 patients were enrolled, among whom 1,321 were diagnosed with pathological vascular tumor thrombus identified through microscopic examination.

Inclusion criteria:

Patients included in this study were those who underwent colorectal surgery between January 1, 2014, and December 31, 2019, with a postoperative pathological diagnosis of colorectal cancer. Only patients with complete clinical and postoperative pathological data were considered eligible.

Exclusion criteria:

Patients were excluded from the study if they had a history of malignancies or were diagnosed with multiple malignancies simultaneously. Additionally, individuals with non-adenocarcinoma histological subtypes of colorectal cancer were also excluded from this study. A small number of patients diagnosed with advanced colorectal cancer who either opted for conservative treatment or discontinued treatment without undergoing surgery were not included in the analysis. Furthermore, patients diagnosed with colorectal cancer at our hospital but who underwent surgery at other institutions were excluded due to incomplete postoperative pathological data.

**Univariate and multivariate Cox regression analysis**

To verify whether the tumor thrombus was an independent prognostic factor, we performed a Cox regression analysis. The variables included in the univariate Cox regression analysis included gender, age, location, intestinal obstruction, positive lymph nodes, lymph nodes, pT, pN, pM, TNM stage, tumor thrombus and tumor size. Significant factors were included in the multivariate Cox regression analysis. The results are shown in a forest diagram.

**Survival analysis**

We applied R packages “survival” (V3.4.0) and “survminer” (V0.4.9) to assess the association between tumor thrombus and clinical outcomes. We used log-rank test to calculate groups’ difference, and Kaplan−Meier (K-M) method implemented in ggsurvplot function to plot survival curves.

**ST samples handling**

Four ST samples, surgically excised between July 2022 and April 2023, were obtained from formalin-fixed, paraffin-embedded (FFPE) blocks of four primary colorectal cancer (CRC) patients with pathologically confirmed tumor thrombus at Xiangya Hospital. We opted to excise the entire tumor thrombus along with the adjacent tumor tissue within a 5mm radius for analysis, ensuring a comprehensive examination of the tumor thrombus and surrounding tissue characteristics. None of the four patients had undergone preoperative chemotherapy or radiotherapy. Their clinical data are available in Table S2. The use of their tissues for this research was fully approved by the Ethic Committee of the Xiangya Hospital of Central South University.

FFPE samples were prepared following the tissue preparation guide protocols, cutting at 5 µm thickness and mounted onto each Visium slide 6.5*6.5 mm2 capture area for the 10X Genomics Visium FFPE spatial gene expression slides. H&E staining was performed concurrently with the Visium FFPE spatial gene expression slides and reagents, all following the guidelines provided by 10X Genomics. Each capture area, measuring approximately 5,000 barcoded spots of 55 μm in diameter, was subjected to RNA probe hybridization, ligation and barcoding to construct Illumina sequencing libraries, using the Visium Spatial Gene Expression Reagent Kits for FFPE according to the 10X Genomics instructions. Libraries were sequenced using the NovaSeq 6000 platform (Illumina) to a depth of approximately 500 million reads per library with 2×150 read length.

**Spatial RNA-seq data processing, visualization and integration**

Raw sequencing reads were processed with Space Ranger (V2.0.1) and aligned to human genome (hg38). Seurat (V4.3.0) was used to process the Space Ranger output files. From the filtered spots, we used the SCTransform function to normalize the data and integrate the expression data from different sections of each patient. We used the FindVariableFeatures function to obtain the top 3,000 highly variable genes (HVGs) from the corrected expression matrix. These HVGs were then centered and scaled using the ScaleData function. Principal component analysis (PCA) was performed on the HVGs using the RunPCA function to reduce dimensionality. After PCA, the main spot clusters were identified using the Louvain-Jaccard graph-based method with the FindNeighbors and FindClusters functions. The clustering resolution parameter was set to 0.20. We then used RunUMAP with 30 dimensions to reduce the high-dimensional data into two dimensions for visualization. For each of the identified clusters, we ran the FindAllMarkers function with default parameters to identify genes that were specifically expressed in each cluster. The significance of the differences in gene expression was determined using the Wilcoxon rank sum test with Bonferroni correction. For the integration of the scRNA-seq dataset with the spatial transcriptomic dataset, we preprocessed the scRNA-seq data as a reference and performed label transfer as outlined in the Seurat protocol. we used the FindTransferAnchors function with default parameters, using SCT as normalization method. Then, the TransferData function (weight.reduction = “pca”, 20 dimensions) was used to annotate spatial regions based on transferred anchors from the scRNAseq reference dataset. For each of the scRNA-seq-derived groups, the procedure generates a probabilistic classification for each position, that is a prediction score. The annotated clusters from the scRNA-seq data set could thus be projected onto the spatial tissue image using SpatialFeaturePlot function.

**ST cell types annotation**

To infer the composition of cell types within each ST spot, which encompasses multiple cells, we employed an integrative approach combining copy number alteration (CNA) analysis and deconvolution methodology. CNA analysis across all ST spots was inferred using the R package "inferCNV" (V1.10.1)^1^, with non-carcinoma regions serving as reference cells due to their presumed absence of CNA. We calculated an average gene expression value over a chromosomal window (default = 100 genes) in carcinoma regions and compared the value to non-carcinoma regions. Carcinoma region spots were annotated as CNA-malignant spots if they had an average CNA score > 1.1 or <0.9.^2^ scRNA-seq data for CRC were obtained from dataset GSE132465, which comprised 23 CRC tissue samples and 10 normal tissue samples from CRC patients. Following data normalization, principal component analysis (PCA), dimensionality reduction and clustering using the R package “Seurat” (V4.3.0), we successfully identified ten distinct cell types based on typical cell markers. Subsequently, these identified cell types served as a reference for deconvolution of spatial transcriptomics spots using the R package “CARD” (V1.1) with min count gene 100 and min count spot 5. Integrating the CNA and deconvolution analysis, the spots identified as CNA-malignant spots through CNA analysis or as malignant epithelial spots through deconvolution, are designated as malignant epithelial cell spots. The remaining spots are classified based on deconvolution results into various cell types, including nonmalignant epithelial cells, fibroblasts, CD4^+^ T cells, CD8^+^ T cells, SMC, endothelial cells, B cells, mast cells and macrophages.

**Malignant epithelial cells correlation matrix**

The Pearson correlation coefficient was employed to assess the association of average expression levels across the entire transcriptome, utilizing the 'cor' function within the R statistical package "stats" (V4.1.2). Subsequently, the correlation values were visualized through the R package "ComplexHeatmap" (V2.10.0).

**DEGs analysis**

Differentially expressed genes (DEGs) testing of two groups with each clusters were performed using the FindMarkers function in “Seurat” (V4.3.0). The significance of the differences in gene expression was determined using the Wilcoxon rank sum test with Bonferroni correction. The DEGs of two groups in each cluster were determined based on following criteria: 1) expressed in more than 10% of the cells within either group or both of the two groups; 2) |log2FC| > 0.25; 3) Wilcoxon rank sum test adjusted p-value < 0.05.

**GO/KEGG enrichment analysis**

Enrichment scores (p-values) for selected numbers of GO/KEGG annotations were calculated by clusterProfiler(v3.14.3) R package with a hyper-geometrical statistical test with a threshold of 0.05, and the Benjamini-Hochberg method was used to estimate the false discovery rate (FDR). Enrichment was calculated for the input DEGs in subcluster. The background was all the genes listed in the database of org.Hs.eg.db.

**Gene set variation analysis (GSVA)**

Pathway analysis was predominantly performed on the HALLMARK described in the Molecular Signatures Database (MSigDB) H collection, exported using the GSEABase package (V1.48.0). To assign pathway activity estimates to individual spot, we applied GSVA^3^ using standard settings, as implemented in the package “GSVA” (V1.34.0). To assess differential

activities of pathways (GSVA) between two clusters, we contrasted the activity scores for each cell using a generalized linear model in limma package (v3.46.0).

**Transcription factor regulons inference**

The regulatory network and activity of regulons were identified using the SCENIC^4^ facilitated by the R package "pySCENIC" (V0.11.2). Genes present in less than 1% of all spots were excluded from analysis. Initially, a co-expression network for transcription factor (TF) targets was constructed utilizing the random forest algorithm via GENIE3. Subsequently, TF binding motifs proximal to transcription start sites in the hg38 reference genome were obtained from cisTarget. A comprehensive TF motif enrichment analysis was performed, and target genes for each TF module were determined using RcisTarget. Moreover, regulon activity was quantified using AUCell, which computes the area under the recovery curve across gene expression rankings for each spot, resulting in the generation of a binary matrix reflecting regulon activity. Differentially activated regulons between TT and NTT were identified through Wilcoxon tests.

**RNA velocity**

To characterize dedifferentiation transitions between different CMS subtypes, we applied “velocyto” (V0.17) tool.^5^ Using the velocyto command line to processed BAM files that generated by Spatial fastq along with their annotations to calculate spliced and unspliced transcript counts for each cell. Subsequently, package “scVelo” (V0.2.5) were performed through dynamics algorithm with default parameter; Cells were embedded into a two-dimensional space using manifold learning techniques UMAP to visualize transitions between CMS subtype cell states.

**CRC stem cell and EMT scores**

For calculation of CRC stem cell and EMT scores in single spot, AddModuleScore was performed. The CRC stem cell signature is a result of four independent CRC research projects.^6-9^ The EMT hallmark gene signature was downloaded from the Molecular Signatures Database (https://www.gsea-msigdb.org/gsea/msigdb/index.jsp).

**Spatial co-localization analysis**

To evaluate the co-localization of diverse cell types within each ST spot, we quantified the Pearson correlation coefficients for each pair of cell types across all spots. Subsequently, to ensure the reliability of our statistical inferences, we employed the Holm correction for multiple comparisons using the R package "psych" (V2.2.5).^10^ We evaluated correlations between pairs using nearest neighbor analysis. For each ST spot, we calculated the normalized gene expression values by considering both the central spot and its six immediately adjacent neighbors.

**Immunofluorescence staining**

Five micrometer thick sections were generated from CRC FFPE blocks. Immunofluorescence staining was performed using a multiplex fluorescent staining kit (AFIHC023 and AFIHC024, AiFang Biological). For the primary CRC slides, antibodies against CD4 (AF20210, AiFang Biological), CD8 (AF20211, AiFang Biological), CD68 (AF20022, AiFang Biological), CD206 (24595, Cell Signaling Technology), FOXP3 (MAB8214, R&D Systems), CD86 (13395-1-AP, Proteintech), and CD11c (GB11059, Servicebio) were used. The slides were washed after incubation with each primary antibody, followed by incubation with the corresponding secondary antibodies and different dyes at room temperature. Fluorescent signal intensity was determined using a Carl Zeiss microscope and processed with ImageJ (NIH).

**qPCR analysis**

Total RNA was extracted from primary CRC tissues using the TRIzol reagent (Invitrogen). Reverse transcription was performed using 1 μg of total RNA and Super Script II (Invitrogen). PCR amplification reactions were conducted in 25 μl reaction volumes containing SYBR Green PCR Master Mix (PE Applied Biosystems), 1 μl of cDNA, and specific amplification primers. GAPDH served as the internal control. The primer sequences are detailed in Table S9.

**Cell-cell interactions**

The spatial receptor-ligand interaction network between different cell type spots were predicted using R package “CellChat” (V2.1.2) with default parameters.^11^ The CellChat analysis was conducted using the complete human built-in interaction database (InteractionDB v2), focusing on pathways that exhibited significant activity with a p-value cutoff of < 0.05. This analysis was performed on data normalized by Seurat's SCT. Cellchat and ggplot2 draw all plot. Receptor-ligand pairs enriched between clusters were shown in Table S6.

**Bulk RNA-seq data**

Bulk RNA-seq data and corresponding clinical data for CRC were accessed from two independent TCGA datasets (TCGA-COAD and TCGA-READ). We excluded samples lacking survival data or status and included 591 tumor samples in the analysis. We also utilized two external validation cohorts: GSE39582^12^ cohort with 566 samples and GSE38832^13^ cohort with 122 samples after removing samples without follow-up.

**Construction of the tumor thrombus gene signature**

A univariate Cox regression analysis was conducted to evaluate the prognostic significance of the 6,262 differentially expressed genes for OS in TCGA-CRC patients. Genes exhibiting a p-value < 0.05 and a HR > 1 were identified as tumor thrombus gene signature. Tumor thrombus scores were calculated for each patient using the ssGSEA method based on R package “GSVA” (V1.46.0). Subsequently, patients were divided into high and low tumor thrombus score groups based on the median score, and the R package “survminer” (V0.4.9) was used for survival analysis of OS based on high and low score groups.

**Immune cells infiltration**

The tumor immune dysfunction and exclusion (TIDE) scores were calculated online (<http://tide.dfci.harvard.edu/>).^14^ The ESTIMATE algorithm^15^ was employed to calculate the scores of stromal and immune cells in the different tumor thrombus groups by the R package “estimate” (V1.0.13). The CIBERSORT algorithm^16^, provided by the R package “CIBERSORT” (0.1.0), was adopted to evaluate the proportion and infiltration of 22 immune cells in TCGA-CRC patients. The Wilcoxon test was applied to analyze the difference in infiltrated immune cells between the high and low score groups.

**Drug sensitivity analysis**

The data in Genomics of Drug Sensitivity in Cancer Phase 2 (GDSC2) from R package “oncoPredict” (V0.2) was employed to predict drug sensitivity between the high and low score groups. Significant differences in IC_50_ between the two groups were evaluated with the Wilcoxon test.

**Statistical analysis**

All statistical analysis were performed using R software and associated R packages. The Wilcoxon test was conducted to compare the differences among distinct groups. All statistical analysis were bilateral, and p-value< 0.05 was considered statistically significant.

References

1. Patel AP, Tirosh I, Trombetta JJ, et al. Single-cell RNA-seq highlights intratumoral heterogeneity in primary glioblastoma. *Science*. 2014;344(6190):1396-1401. doi:10.1126/science.1254257

2. Zheng Y, Carrillo-Perez F, Pizurica M, et al. Spatial cellular architecture predicts prognosis in glioblastoma. *Nat Commun*. 2023;14(1):4122. doi:10.1038/s41467-023-39933-0

3. Hanzelmann S, Castelo R & Guinney J. GSVA: gene set variation analysis for microarray and RNA-seq data. *BMC Bioinformatics*. 2013;14(1471-2105 (Electronic)):7. doi:10.1186/1471-2105-14-7

4. Aibar S, Gonzalez-Blas CB, Moerman T, et al. SCENIC: single-cell regulatory network inference and clustering. *Nat Methods*. 2017;14(11):1083-1086. doi:10.1038/nmeth.4463

5. La Manno G, Soldatov R, Zeisel A, et al. RNA velocity of single cells. *Nature*. 2018;560(7719):494-498. doi:10.1038/s41586-018-0414-6

6. Merlos-Suarez A, Barriga FM, Jung P, et al. The intestinal stem cell signature identifies colorectal cancer stem cells and predicts disease relapse. *Cell Stem Cell*. 2011;8(5):511-524. doi:10.1016/j.stem.2011.02.020

7. Abbasian M, Mousavi E, Arab-Bafrani Z, et al. The most reliable surface marker for the identification of colorectal cancer stem-like cells: A systematic review and meta-analysis. *J Cell Physiol*. 2019;234(6):8192-8202. doi:10.1002/jcp.27619

8. Lugli A, Iezzi G, Hostettler I, et al. Prognostic impact of the expression of putative cancer stem cell markers CD133, CD166, CD44s, EpCAM, and ALDH1 in colorectal cancer. *Br J Cancer*. 2010;103(3):382-390. doi:10.1038/sj.bjc.6605762

9. Munro MJ, Wickremesekera SK, Peng L, et al. Cancer stem cells in colorectal cancer: a review. *J Clin Pathol*. 2018;71(2):110-116. doi:10.1136/jclinpath-2017-204739

10. Wang Y, Liu B, Min Q, et al. Spatial transcriptomics delineates molecular features and cellular plasticity in lung adenocarcinoma progression. *Cell Discov*. 2023;9(1):96. doi:10.1038/s41421-023-00591-7

11. Jin S, Guerrero-Juarez CF, Zhang L, et al. Inference and analysis of cell-cell communication using CellChat. *Nat Commun*. 2021;12(1):1088. doi:10.1038/s41467-021-21246-9

12. Marisa L, de Reynies A, Duval A, et al. Gene expression classification of colon cancer into molecular subtypes: characterization, validation, and prognostic value. *PLoS Med*. 2013;10(5):e1001453. doi:10.1371/journal.pmed.1001453

13. Tripathi MK, Deane NG, Zhu J, et al. Nuclear factor of activated T-cell activity is associated with metastatic capacity in colon cancer. *Cancer Res*. 2014;74(23):6947-6957. doi:10.1158/0008-5472.CAN-14-1592

14. Jiang P, Gu S, Pan D, et al. Signatures of T cell dysfunction and exclusion predict cancer immunotherapy response. *Nat Med*. 2018;24(10):1550-1558. doi:10.1038/s41591-018-0136-1

15. Yoshihara K, Shahmoradgoli M, Martínez E, et al. Inferring tumour purity and stromal and immune cell admixture from expression data. *Nature Communications*. 2013;4(1):doi:10.1038/ncomms3612

16. Newman AM, Liu CL, Green MR, et al. Robust enumeration of cell subsets from tissue expression profiles. *Nat Methods*. 2015;12(5):453-457. doi:10.1038/nmeth.3337
