# Supplementary material for: Spatial transcriptomics deciphers the immunosuppressive microenvironment in colorectal cancer with tumour thrombus
Source: Clin Transl Med. 2024 Dec 1;14(12):e70112. doi: 10.1002/ctm2.70112 (PMC11608866; doi:10.1002/ctm2.70112)
Supplement: Supplementary file 2 — Supporting Information [file CTM2-14-e70112-s002.doc]

Supplementary figures

**Spatial Transcriptomics Deciphers the Immunosuppressive Microenvironment in Colorectal Cancer with Tumor Thrombus**

Heming Ge, Zhengda Pei, Zhongyi Zhou, Qian Pei, Cenap Güngör, Linyi Zheng, Wei Liu, Fengyuan Li, Jingxuan Zhou, Yao Xiang, Haiping Pei, Yuqiang Li, Wenxue Liu

**This file includes:**

Figure S1 - S12


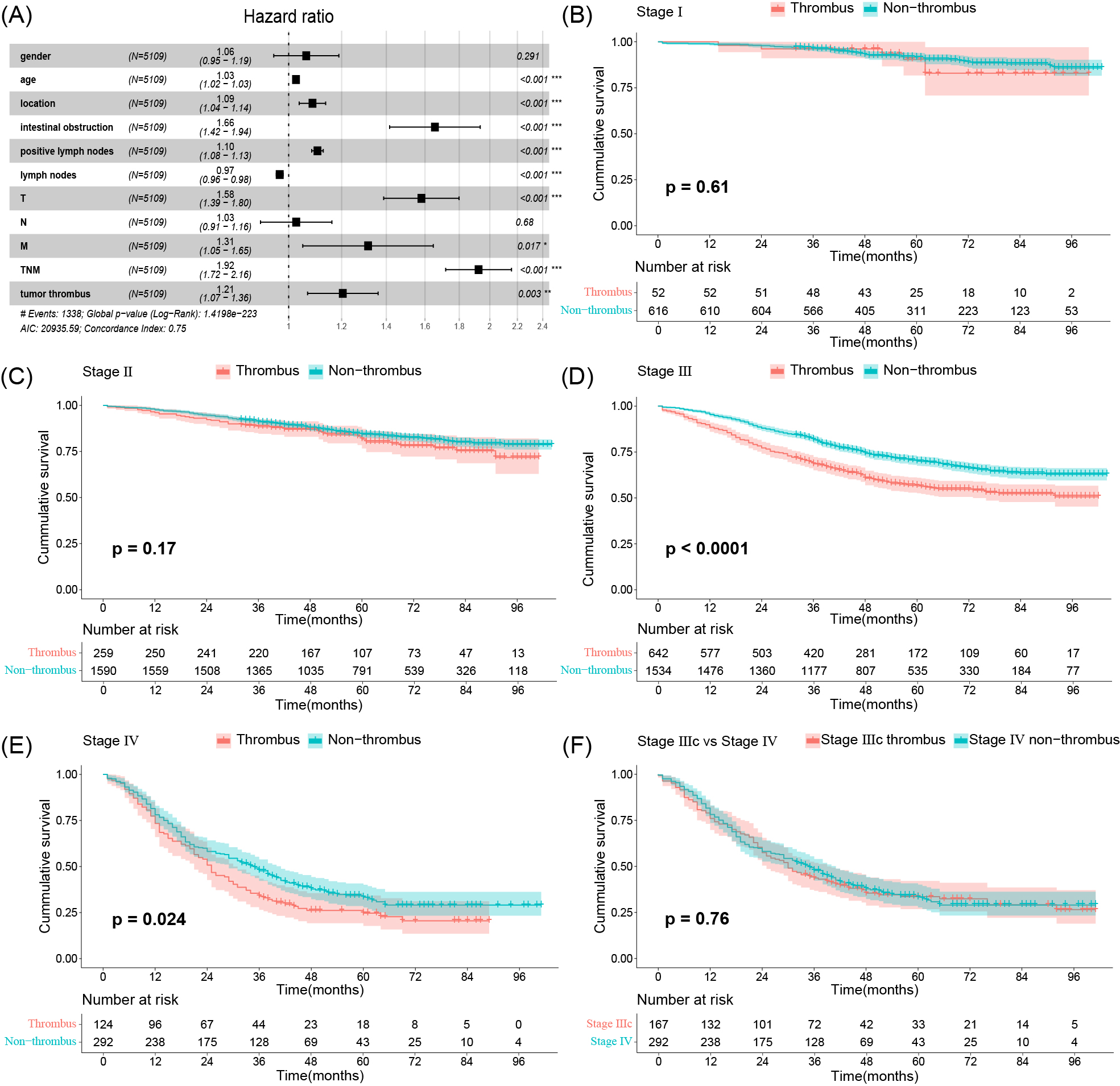


**Figure S1**

CRC tumor thrombus clinicopathological characteristics and prognosis. (A) Multivariate Cox analysis of clinicopathological characteristics. Tumor thrombus served as an independent prognostic factor with a higher HR than that of positive lymph nodes. (B-F) Survival curves for CRC patients across different stages, comparing survival between those with and without tumor thrombus in Stage Ⅰ (B), Stage Ⅱ (C), Stage Ⅲ (D) and Stage Ⅳ (E), and comparing Stage Ⅲc with tumor thrombus to Stage Ⅳ without tumor thrombus (F).


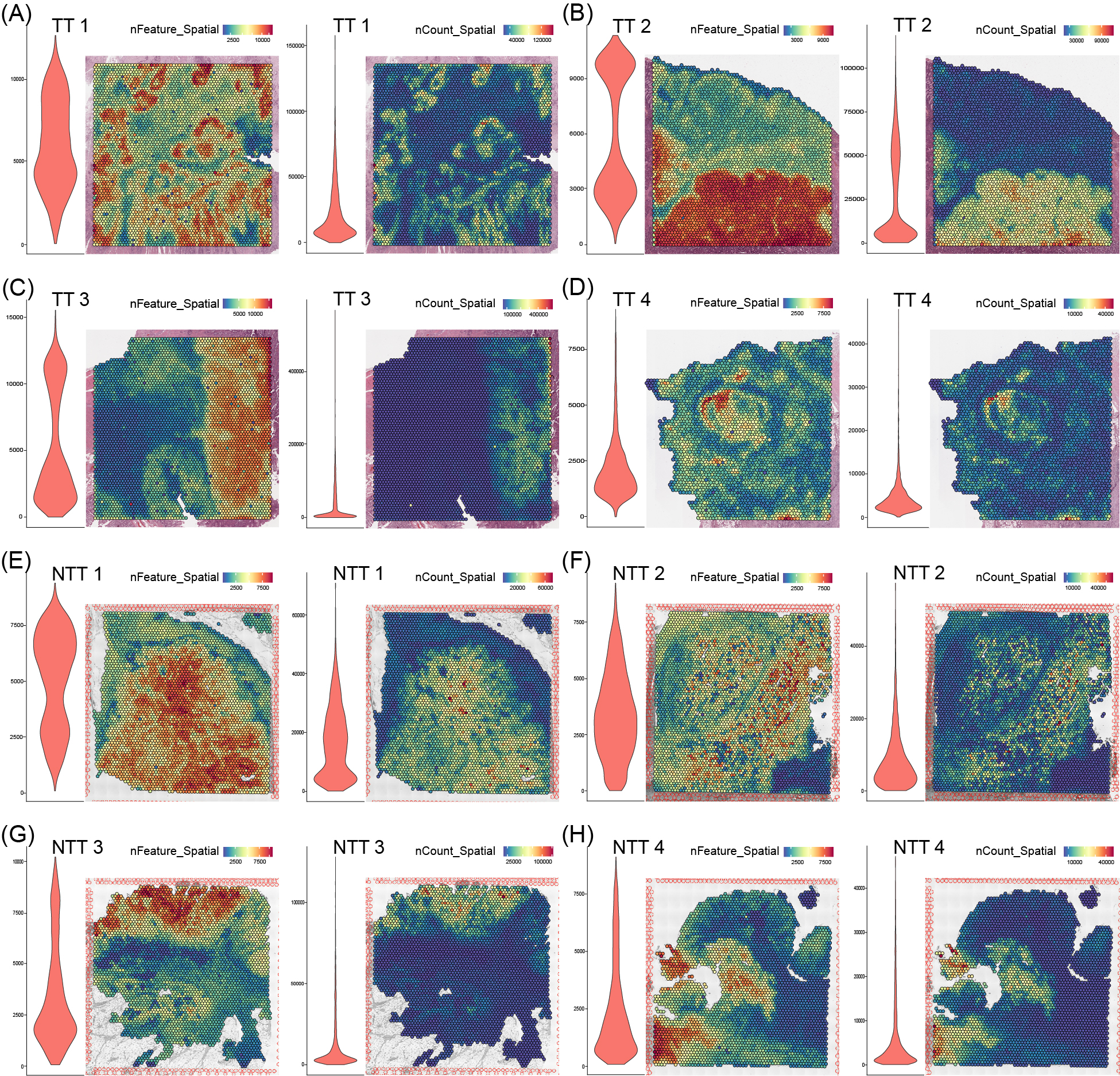


**Figure S2**

Quality control of spatial transcriptomics. The numbers of gene features and UMI counts across all samples. TT, tumor thrombus; NTT, non-tumor thrombus.


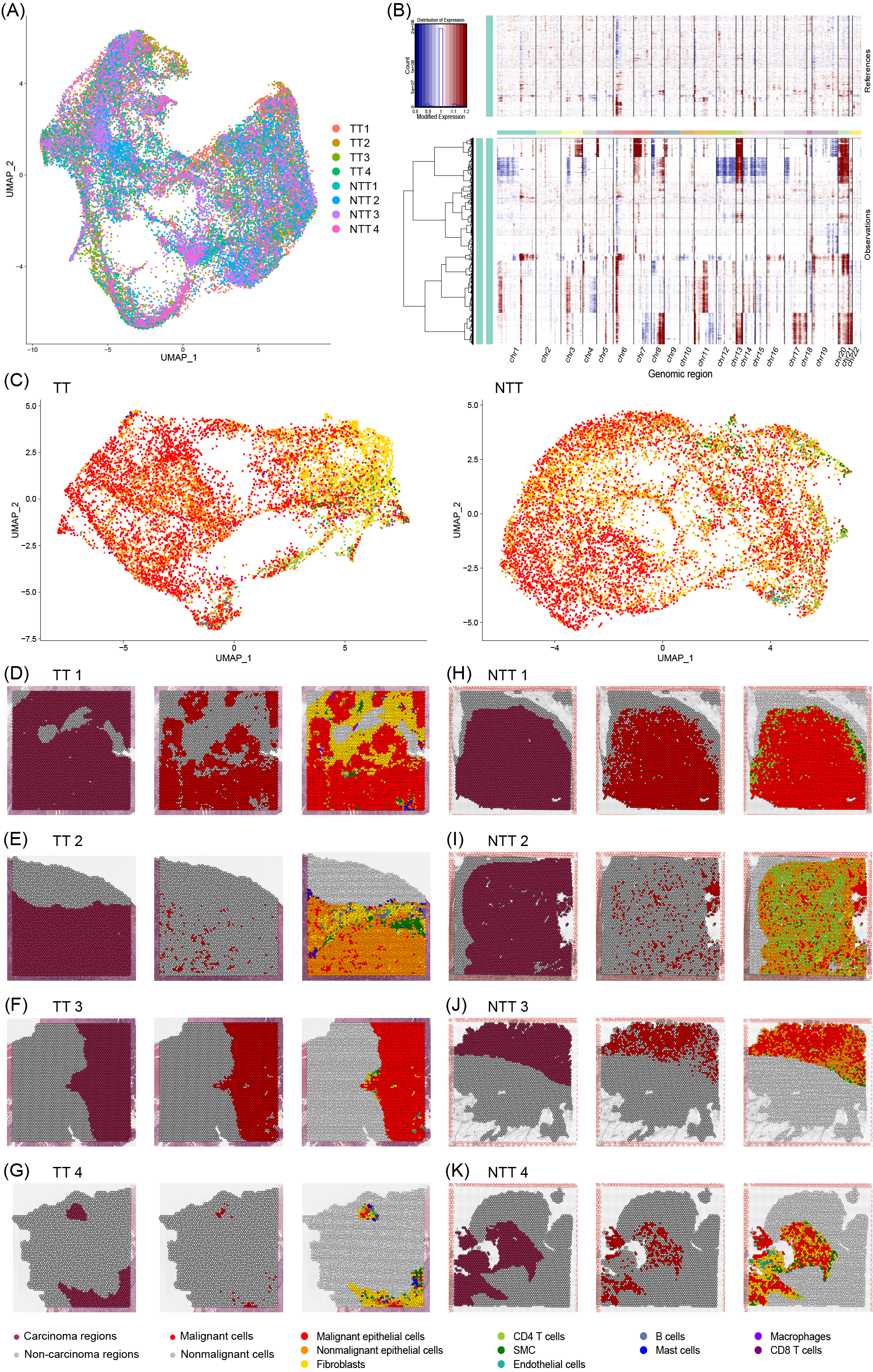


**Figure S3**

Identification of cell type spots in ST samples. (A) UMAP visualization of spot clusters. Each dot represents a spot, and the spot was colored by the originating samples. (B) Heatmaps showing inferred CNAs across different chromosomes in all spots. (C) Annotation of ST spots in carcinoma regions categorized by different groups. (D-K) Spatial distribution of carcinoma regions and various cell type spots in TT and NTT samples. TT, tumor thrombus; NTT, non-tumor thrombus; SMC, smooth muscle cells.


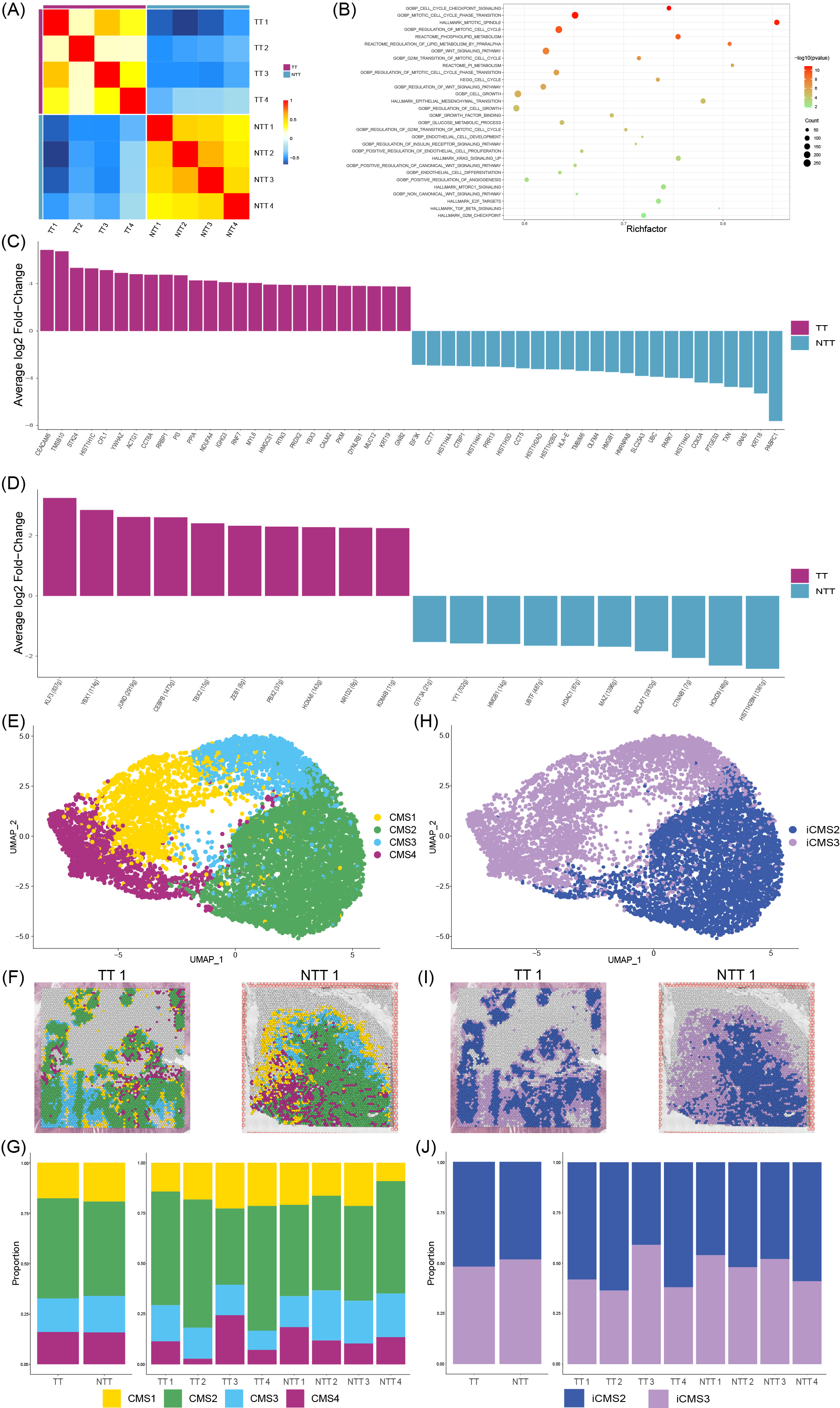


**Figure S4**

Functional and molecular characterization of malignant epithelial cells. (A) Gene expression Pearson correlation matrix across malignant epithelial cells. (B) Pathways enrichment of malignant epithelial cells in TT samples. (C, D) Average logFC for the top 25 genes (C) and top 10 transcription factors (D) significantly differentially expressed between all malignant epithelial cells of TT and NTT samples. (E) UMAP plot displaying the distribution of CMS subtypes across TT and NTT samples. (F) Spatial distribution of different CMS subtypes within TT1 and NTT1 samples. (G) Proportion of different CMS subtypes within the TT and NTT samples. (H) UMAP plot displaying the distribution of iCMS2 and iCMS3 across TT and NTT samples. (I) Spatial distribution of iCMS2 and iCMS3 within TT1 and NTT1 samples. (J) Proportion of iCMS2 and iCMS3 subtypes within the TT and NTT samples.


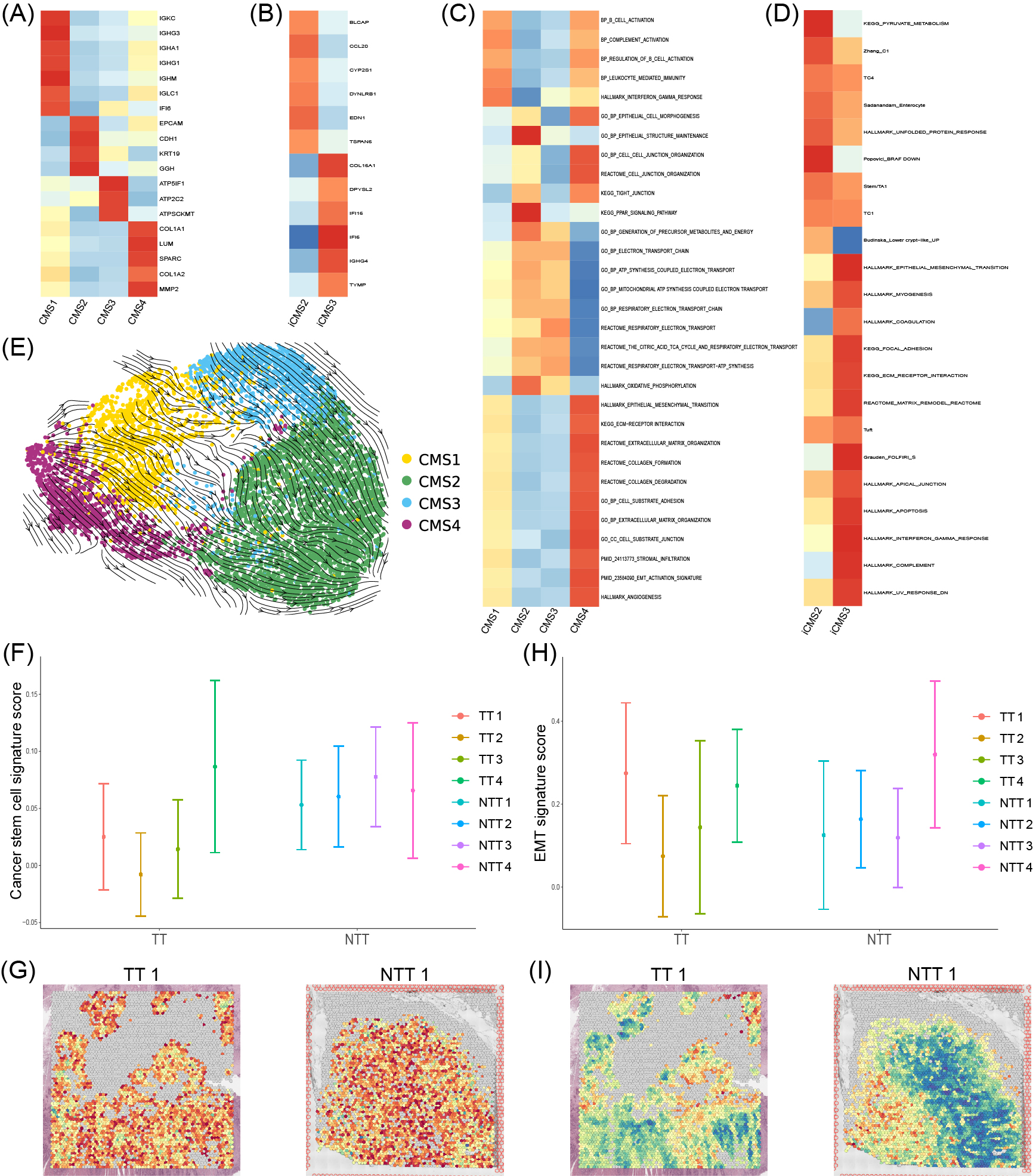


**Figure S5**

Transcriptional alterations and molecular diversities of malignant epithelial cells. (A, B) Differential gene expression in CMS subtypes (A) and iCMS subtypes (B). (C, D) Enrichment pathways across CMS subtypes (C) and iCMS subtypes (D). (E) UMAP plot of spatial cell type spots with RNA velocity streams, colored based on CMS subtypes. (F) Comparative expression of CRC cancer stem cell signature score in malignant epithelial cells across TT and NTT samples. (G) Spatial plots of CRC cancer stem cell signature score in TT and NTT samples. (H) Comparative expression of EMT signature score in malignant epithelial cells across TT and NTT samples. (I) Spatial plots of EMT signature score in TT and NTT samples. TT, tumor thrombus; NTT, non-tumor thrombus; CMS, consensus molecular subtypes.


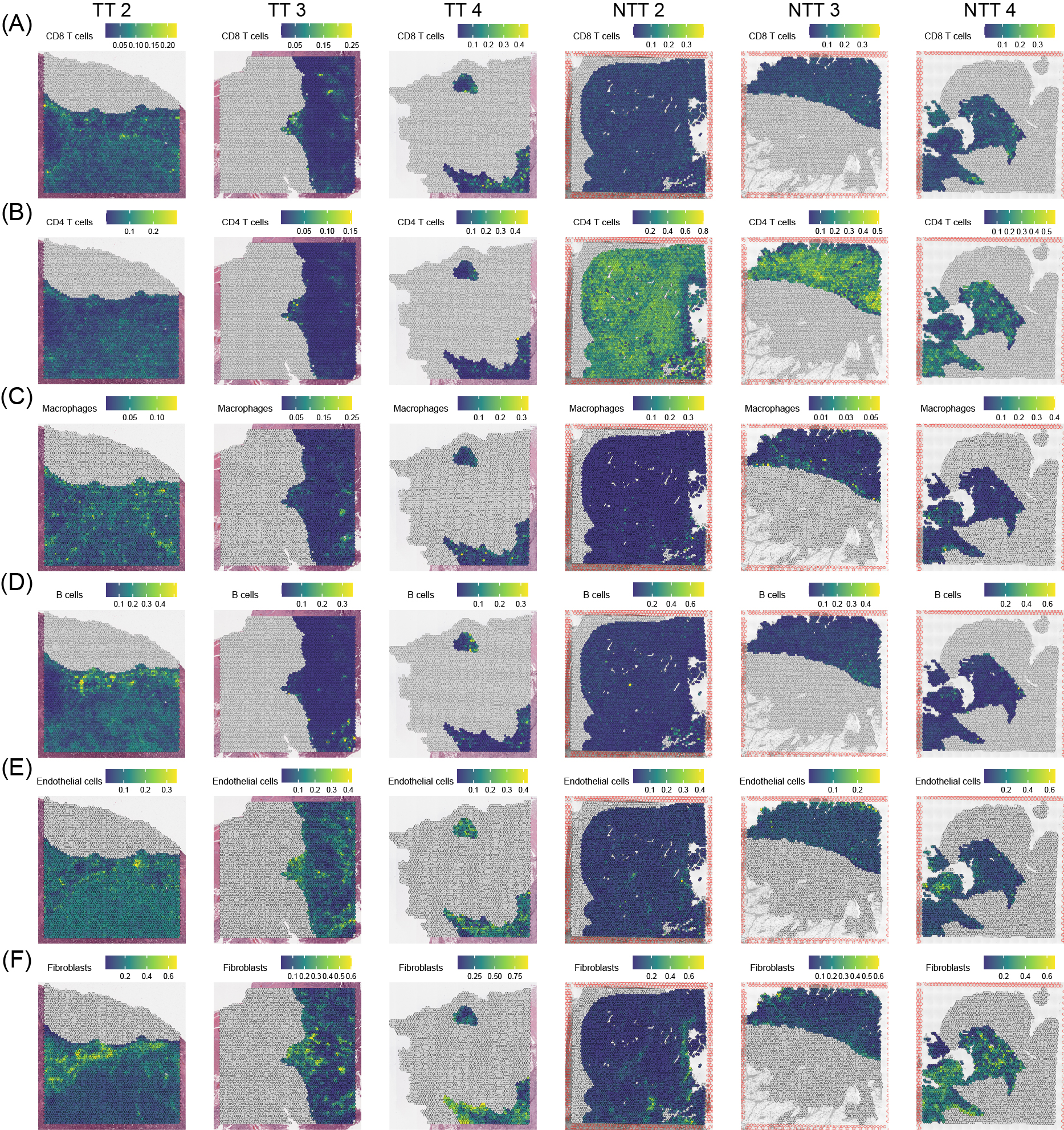


**Figure S6**

Spatial plots of CD8^+^ T cells (A), CD4^+^ T cells (B), macrophages (C), B cells (D), endothelial cells (E) and fibroblasts (F) across TT and NTT samples. TT, tumor thrombus; NTT, non-tumor thrombus; SMC, smooth muscle cells.


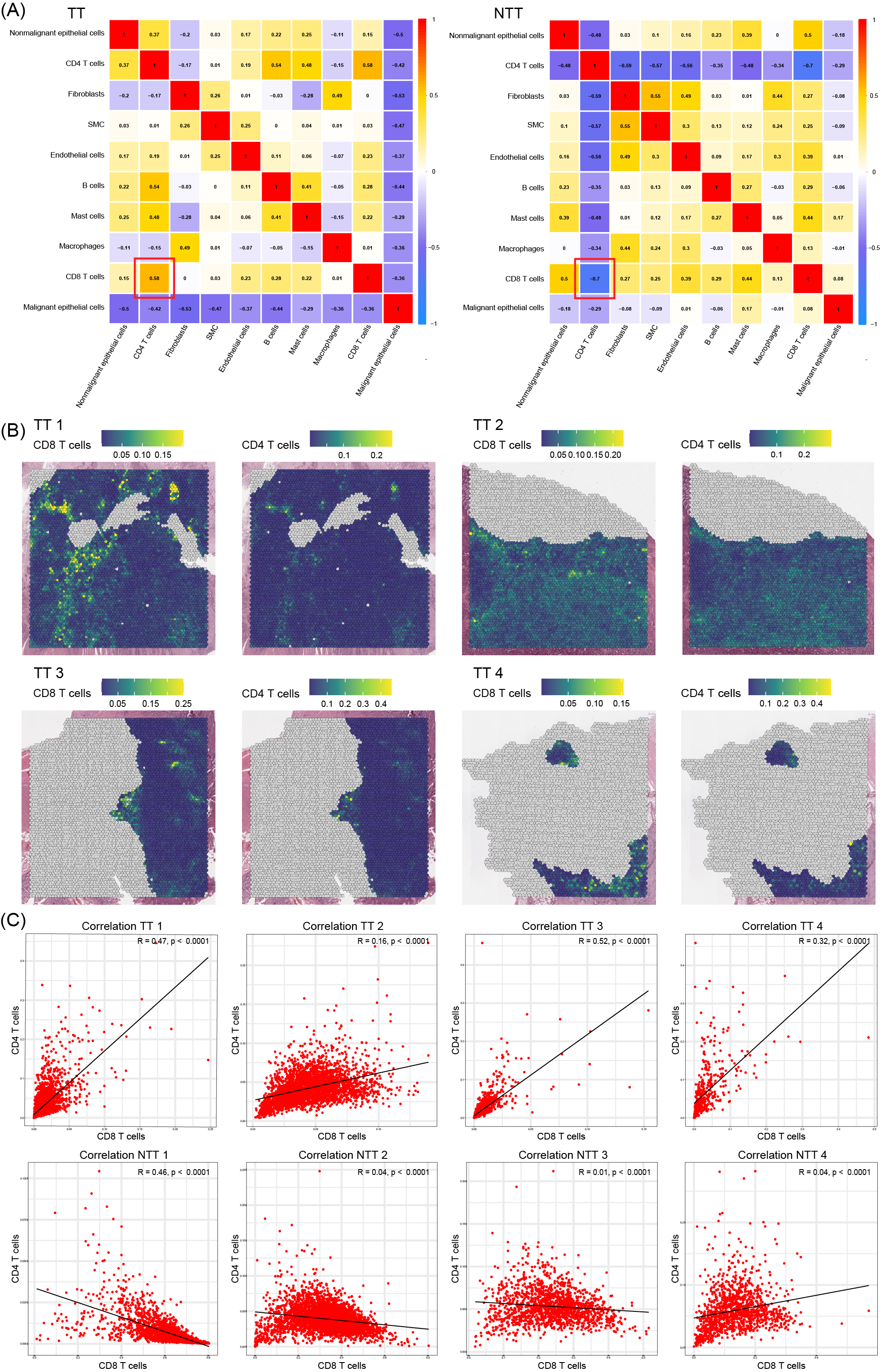


**Figure S7**

Analysis of immune cell dynamics in TT and NTT samples. (A) Spatial Pearson correlation coefficient heatmaps of pairwise cell types. (B) Spatial co-localization pattern of CD4^+^ and CD8^+^ T cells in TT samples. (C) The Pearson correlation of CD4^+^ and CD8^+^ T cells in all spots within TTs and NTTs. TT, tumor thrombus; NTT, non-tumor thrombus; SMC, smooth muscle cells.


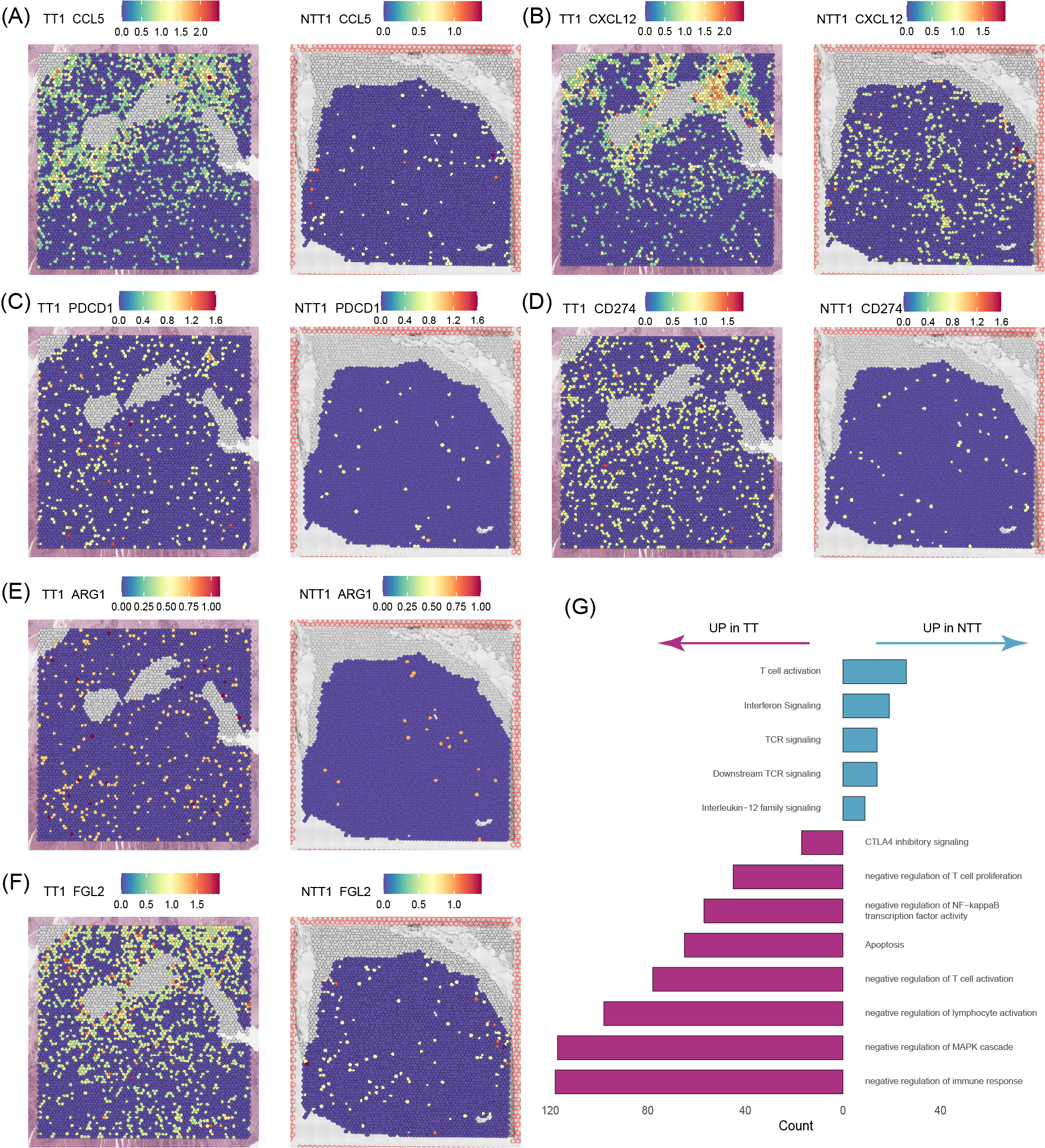


**Figure S8**

Immune and immunosuppressive profiling of CRC tumor thrombus. Spatial plots of gene expression of CCL5 (A), CXCL12 (B), PDCD1 (C), CD274 (D), ARG1 (E) and FGL2 (F) within TT1 and NTT1 samples. (G) Pathways enrichment of CD8^+^ T cells in TTs and NTTs. TT, tumor thrombus; NTT, non-tumor thrombus.


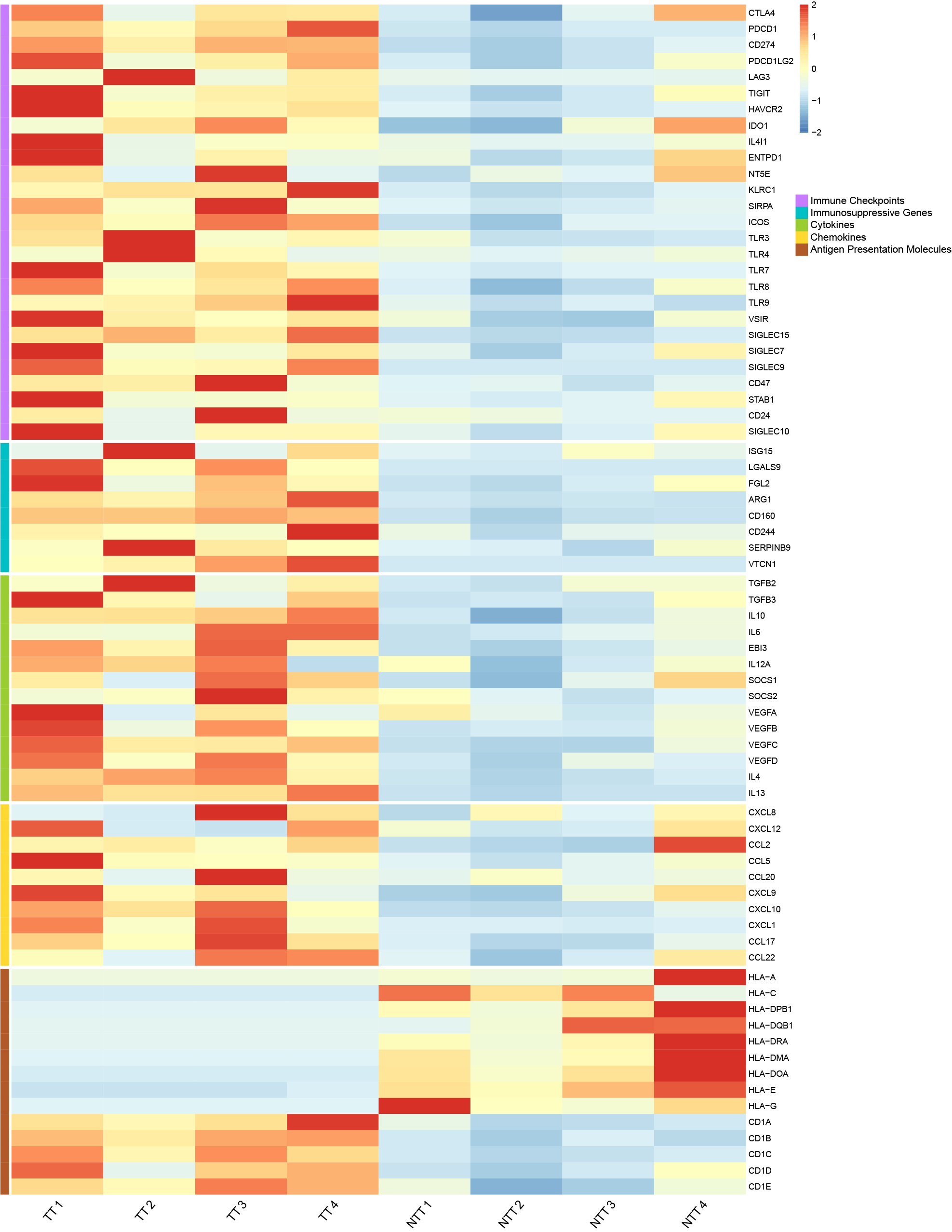


**Figure S9**

Heatmap of immune-related gene expression in CRC tumor thrombus microenvironments. TT, tumor thrombus; NTT, non-tumor thrombus.


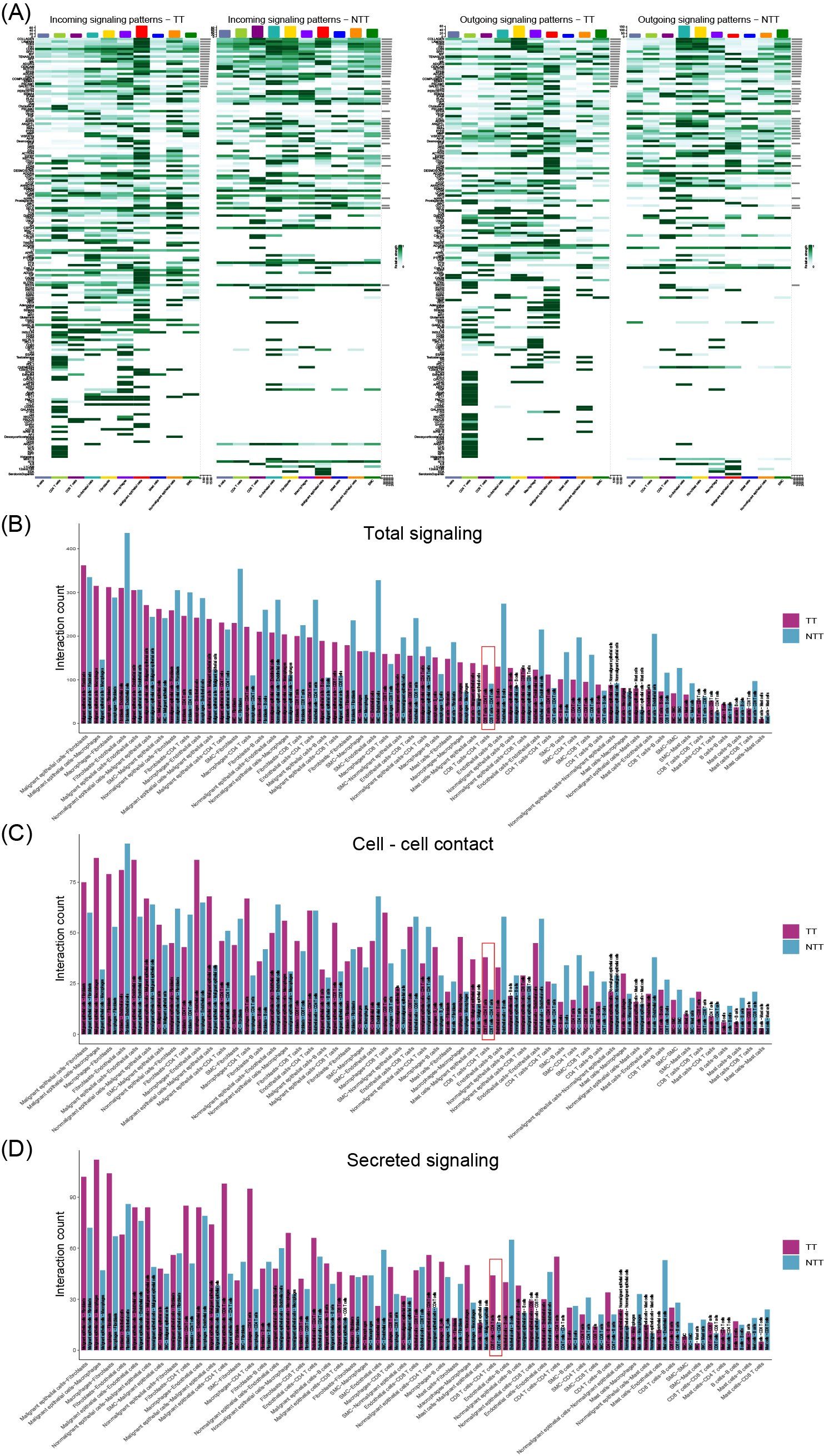


**Figure S10**

Dynamics of intercellular communication in tumor thrombus microenvironments. (A) Total number of intercellular signaling events quantified by receptor-ligand pairs across different cell types in TT and NTT samples. (B-D) Bar chart illustrated the total signaling events (B), cell-cell contact events (C) and secreted signaling events (D) categorized by cell types within TT and NTT samples. Abbreviations: TT tumor thrombus, NTT non-tumor thrombus.


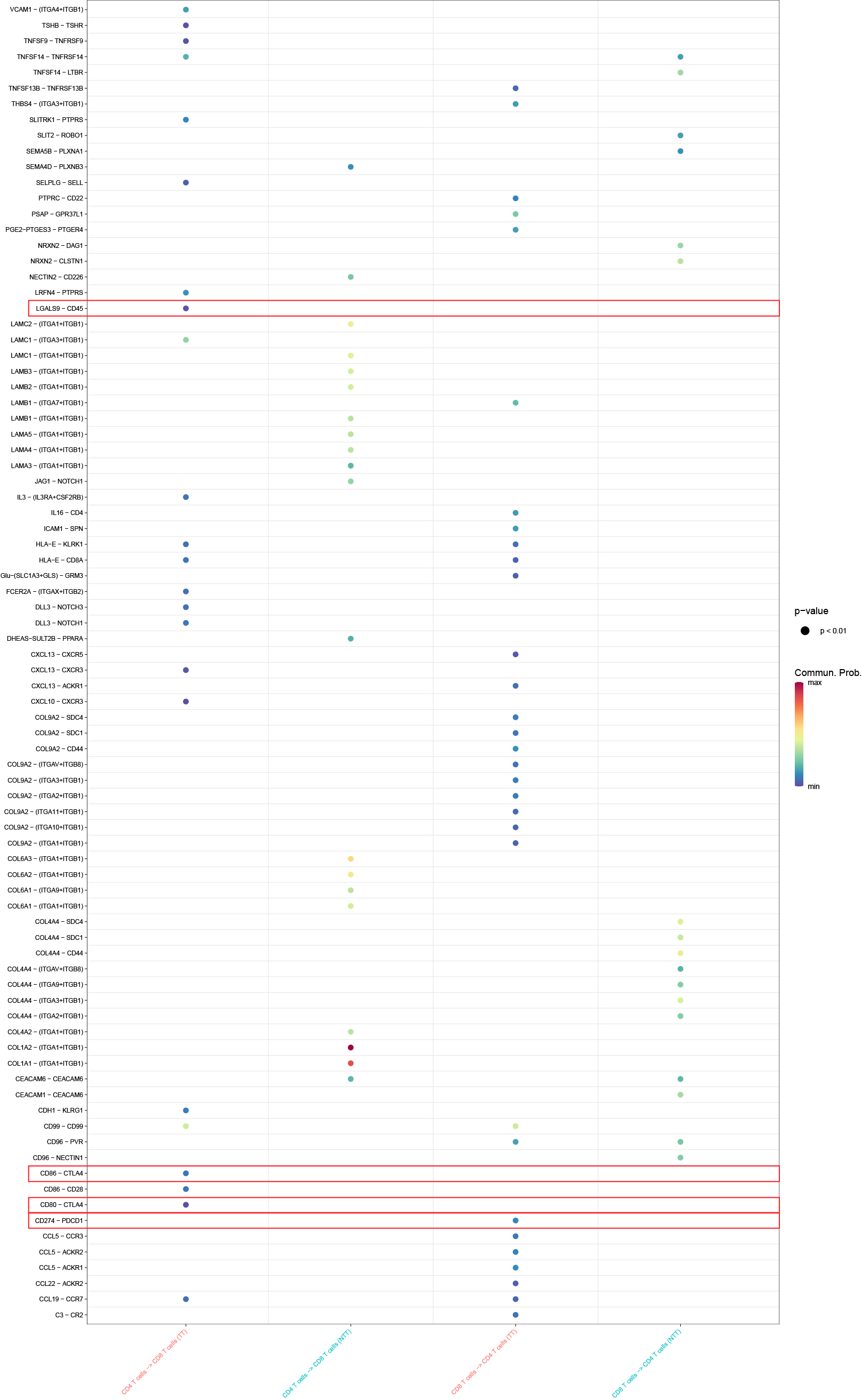


**Figure S11**

Intercellular communication between CD4^+^ and CD8^+^ T cells in the CRC tumor thrombus microenvironments. TT, tumor thrombus; NTT, non-tumor thrombus.


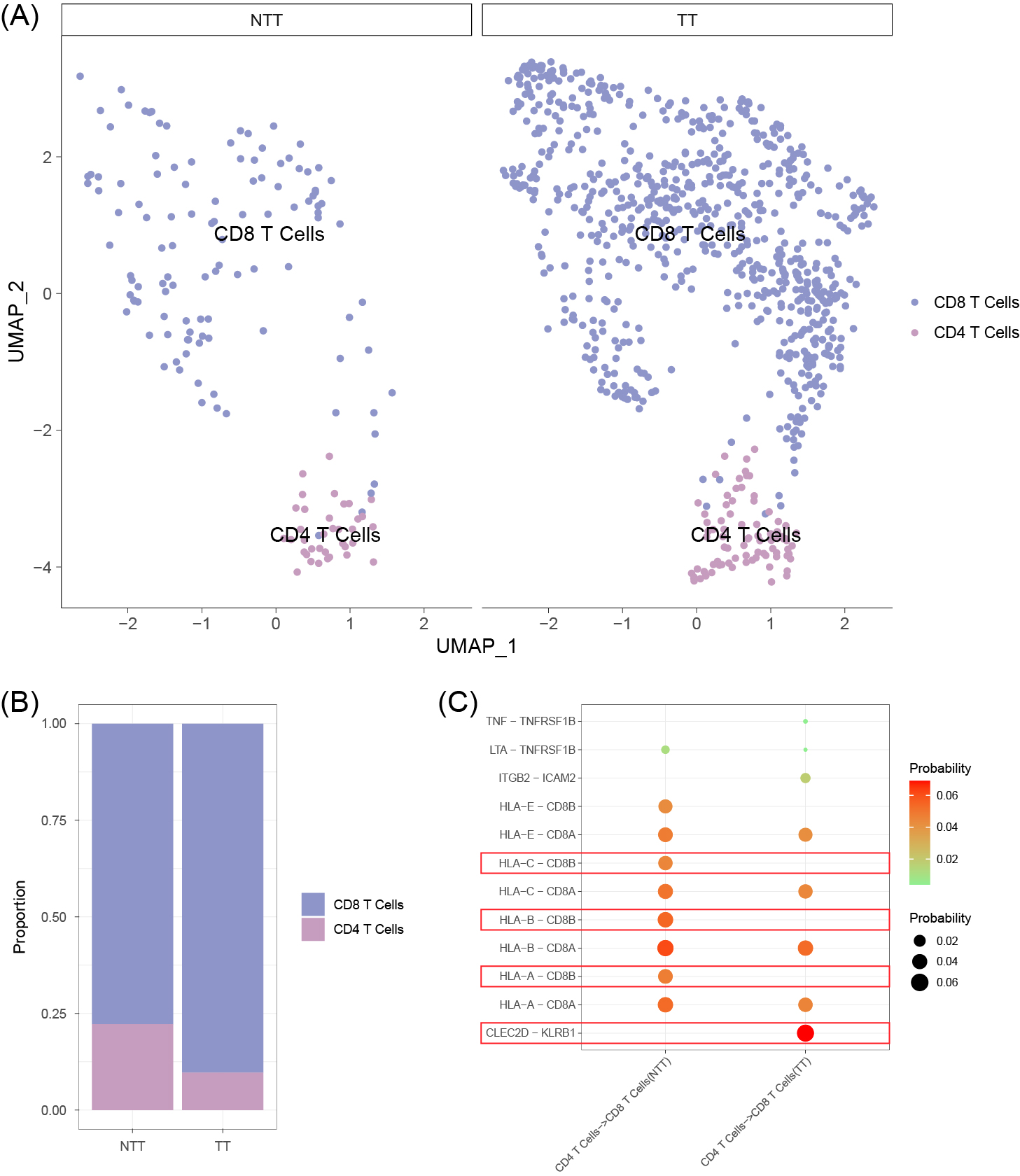


**Figure S12**

Association of CD4^+^ and CD8^+^ T cells between TT and NTT groups in hepatocellular carcinoma single-cell samples. (A) UMAP plots showing the clustering of CD4^+^ and CD8^+^ T cells in both TT and NTT groups. (B) Proportion of CD4^+^ and CD8^+^ T cells in TT and NTT groups. (C) Intercellular communication between CD4^+^ and CD8^+^ T cells in the liver cancer thrombus microenvironments. Inhibitory receptor-ligand pair CLEC2D - KLRB1 was highly expressed in the TT group, while activating receptor-ligand pairs HLA-C - CD8B, HLA-B - CD8B, and HLA-A - CD8B were highly expressed in the NTT group.TT, tumor thrombus; NTT, non-tumor thrombus.


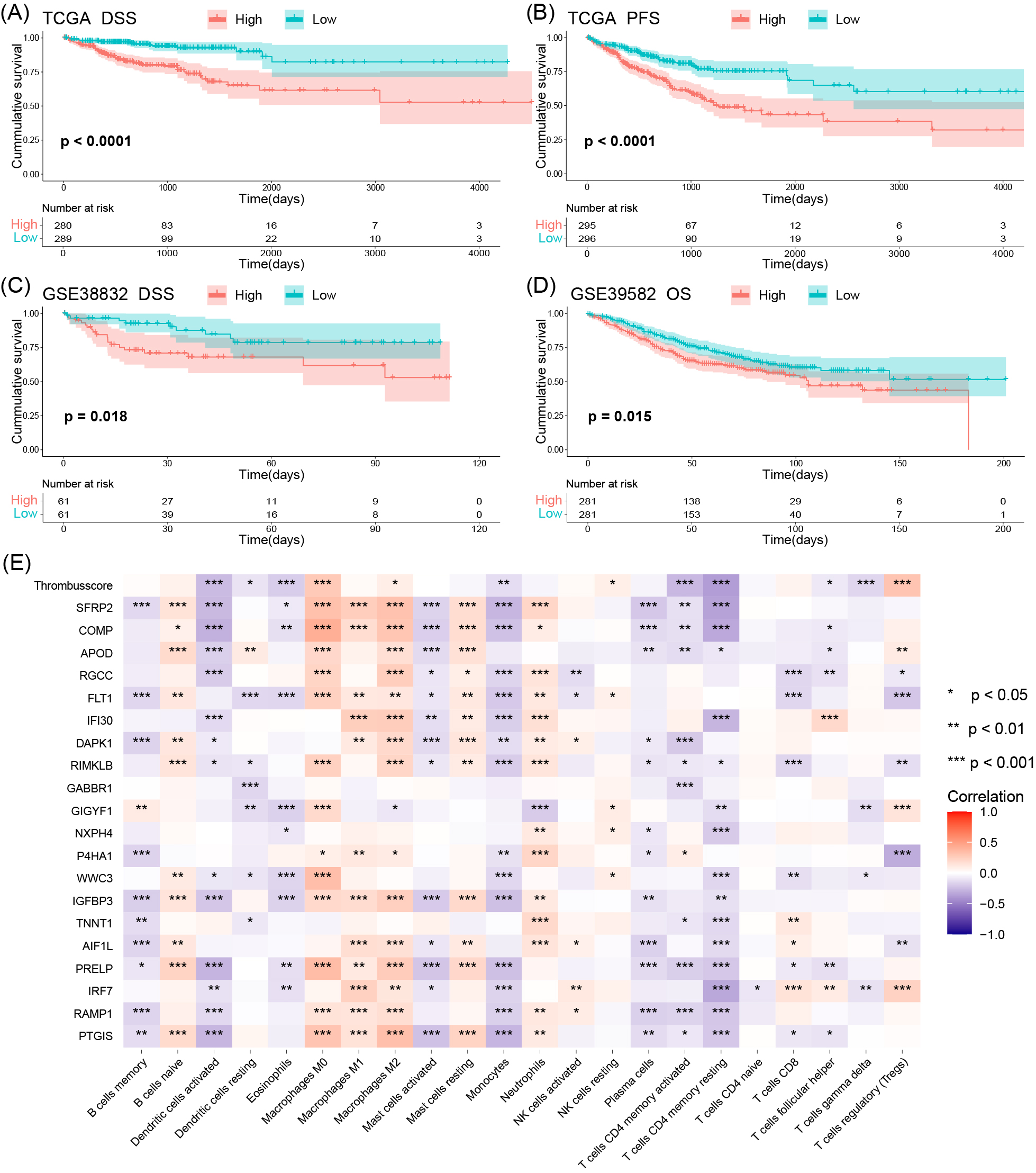


**Figure S13**

Prognosis of the tumor thrombus gene signature and its association with immune cell infiltration in CRC. (A-D) Survival Curves of DSS (A) and PFS (B) in TCGA-CRC dataset. Survival Curves of DSS in dataset GSE38832 (C) and OS in dataset GSE39582 (D). (E) Correlation between hub tumor thrombus genes and 22 immune-related cells. OS, overall survival; DSS, disease specific survival; PFS, progression free survival.
